# A comprehensive comparison between camelid nanobodies and single chain variable fragments

**DOI:** 10.1186/s40364-021-00332-6

**Published:** 2021-12-04

**Authors:** Yasaman Asaadi, Fatemeh Fazlollahi Jouneghani, Sara Janani, Fatemeh Rahbarizadeh

**Affiliations:** 1 Department of Biotechnology, College of Science, University of Tehran, Tehran, Iran; 2 Department of Cell & Molecular Biology, Faculty of Life Sciences and Biotechnology, Shahid Beheshti University, Tehran, Iran; 3 Department of Medical Biotechnology, Faculty of Medical Sciences, Tarbiat Modares University, Tehran, Iran; 4 Research and Development Center of Biotechnology, Tarbiat Modares University, Tehran, Iran

**Keywords:** Single-chain variable fragment (scFv), camelid VHH, nanobody, single-domain antibody

## Abstract

By the emergence of recombinant DNA technology, many antibody fragments have been developed devoid of undesired properties of natural immunoglobulins. Among them, camelid heavy-chain variable domains (VHHs) and single-chain variable fragments (scFvs) are the most favored ones. While scFv is used widely in various applications, camelid antibodies (VHHs) can serve as an alternative because of their superior chemical and physical properties such as higher solubility, stability, smaller size, and lower production cost. Here, these two counterparts are compared in structure and properties to identify which one is more suitable for each of their various therapeutic, diagnosis, and research applications.

## Background

Antibodies (Abs) are distinguished binding tools for targeting almost any biomarker specifically. Although the inherent high affinity and specificity of immunoglobulins (Ig) is achieved by series of somatic hyper mutations and affinity maturation processes in the B cells, the recombinant DNA technology facilitates in-vitro production of various antibodies for a diverse set of targets [1]. Up to date, with about 100 FDA-approved antibodies in the market [2], the monoclonal antibody is a 145 billion dollar industry with 11% growth rate [3].

In structure, immunoglobulin (Ig) consists of two separate regions that can be dissociated by proteolytic cleavage with papain and pepsin; namely Fragment antigen-binding (Fab) domain and fragment crystallizable (Fc) region. While Fc region initiates biological processes upon antigen binding, Fab is responsible for antigen recognition, and the binding specificity of the whole Ig molecule is solely dependent on this domain, especially the two variable domains on the top-variable heavy chain (VH) and variable light chain (VL) [4]. This modular structure of immunoglobulin enabled scientists to introduce many structural modifications on the structure of Abs to improve their performance, by means of protein engineering and recombinant DNA technology. Smaller antibody fragments (such as Fab, scFv, diabodies, triabodies, mini bodies, and single-domain antibodies) are among these modified structures, designed to be reliable alternatives for conventional antibodies. Their smaller size, superior properties, and ease of manufacturing while retaining the targeting specificity of the whole Ig molecule make them perfect tools for diagnosis and clinical applications [5, 6].

Among all the engineered and recombinant antibody formats, single-chain variable fragments (scFvs) and camelid heavy-chain variable domains (VHHs) - also known as nanobodies- are the most popular ones. Previously scientists considered single-chain variable fragment (scFv) -composed of VH and VL- as the smallest antibody fragment with the same antigen-binding specificity to the whole Ig molecule. However, the discovery of camelid VHH [7] and shark variable new antigen receptor (VNAR) [8] demonstrated that a single V-like domain can retain the affinity of a whole antibody molecule [9]. Due to the broad and similar applications of scFv and VHH, this article aims to review the differences of these two antibody fragments in structure and function to illustrate whether the superior properties of nanobodies can make them a capable alternative for scFvs or not.

## Nanobody and scFv in structure

**Fig. 1:**
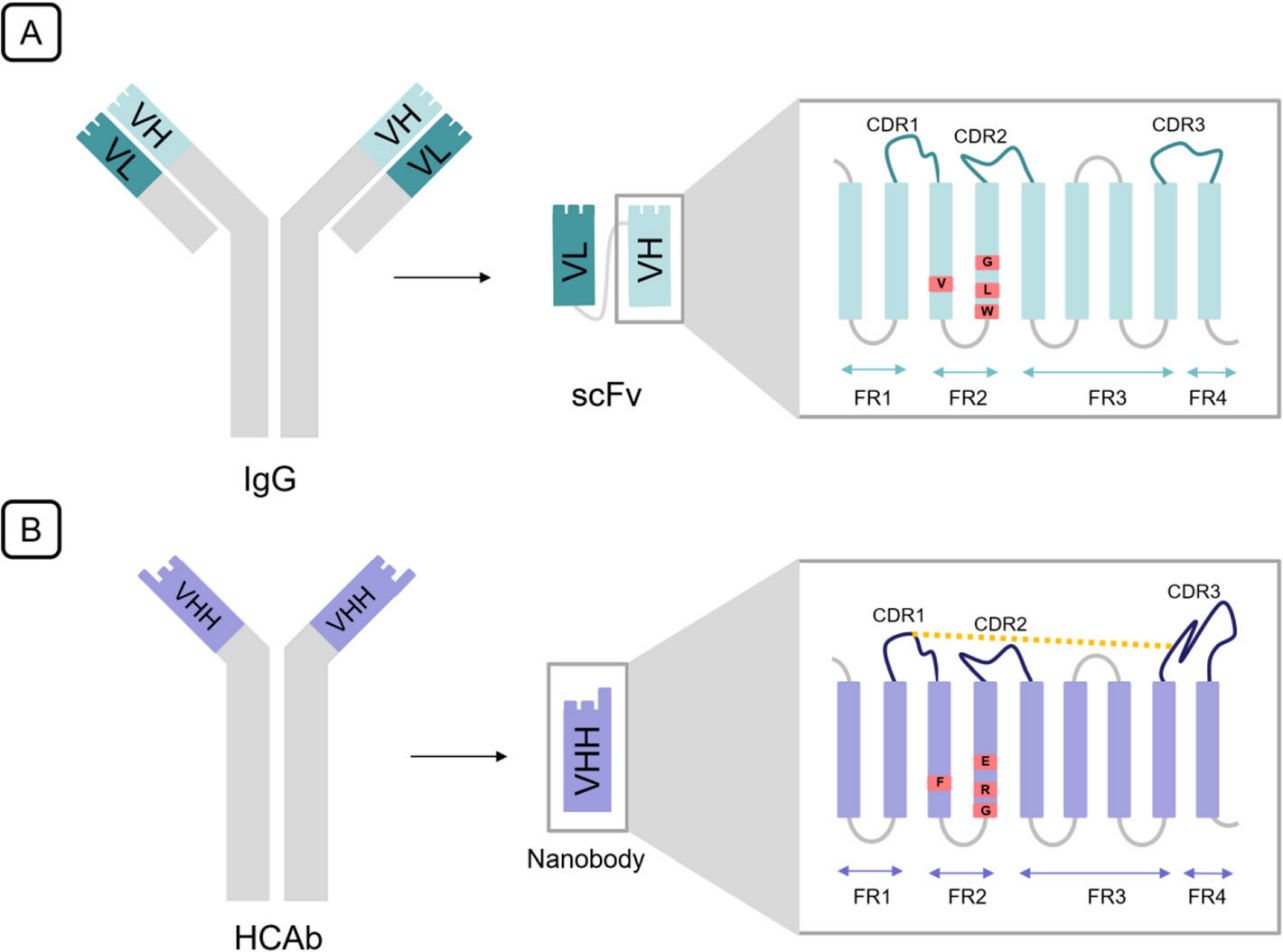
The differences of scFv and nanobody in structure. **A** conventional IgG structure, scFv structure composed of VH and VL of antibody joined with a flexible linker, the detailed structure of VH in scFv. **B** camel HCAb structure, nanobody that is derived from camelid VHH, the detailed structure of VHH

As stated previously, the variable domains of Fab are responsible for the binding specificity of the whole antibody. Therefore, the smallest unit of Ig with antigen-binding activity is the fragment variable or Fv in which the two variable domains (VH and VL) connect with a disulfide bond. ScFv is an engineered form of Fv that, instead of a disulfide bond, the two variable domains are joined together by a flexible linker (Fig. 1). The length and amino acid composition of this linker play an important role in correct folding of the protein [10], and it is typically 10-25 amino acid long with Glu Lys stretches to increase the solubility and Gly Ser stretches for the flexibility of the final protein [11, 12]. Within each of the two variable domains of the scFv, there are three hyper variable domains or complementary determining regions (CDRs) that are linked together with framework regions (FRs). While the CDRs are responsible for antigen binding, and their structure is complementary to the epitope, the remainder of the variable domains (FRs) acts as a scaffold and has inconsiderable variability compared to CDRs. Interestingly, the contribution of each CDR in antigen binding is different. For instance, the CDR3 in the heavy chain has a critical role by 29% contribution in binding specificity while the involvement of CDR2L is just 4% [13].

The higher contribution of the VH in antigen binding raised a research hypothesis that whether a single heavy chain can retain the parent Ab's binding affinity. In seminal studies, mouse single variable domains were investigated for their functional activity [14]. However, their troublesome properties, such as low affinity, poor solubility, and higher production cost, hampered their broader development. The discovery of heavy chain only antibodies (HCAbs) in camelids [7] and immunoglobulin new antigen receptor (IgNAR) in cartilaginous fish [15] was a new beginning in single domain antibody development. The antigen-binding domain of these specific immunoglobulins (VHH and V-NAR) is a high affinity single V-like domain that has evolved to be devoid of the disadvantages of previous single domain fragments. These superior properties result from major adaptations in sequence and structure [16–19]. As camelid nanobodies have easier handling, more robust antibody responses [17, 20], and higher yield in recombinant expression [21] than shark V-NARs, VHH fragments are more frequently used and will be the focus of this review.

Similar to VH, camelid VHHs comprise nine beta-strands forming a typical IgV fold; however, VL loss caused notable differences between these two fragments, especially in FR2 and hypervariable loops. In the conventional VH region, the FR2 consist of four highly conserved hydrophobic amino acids (Val37, Gly44, Leu45, and Trp47) that in contribution with Gln39, Gly44, Tyr91, and Trp103 form a conserved hydrophobic interface of ~700 Å^2^ to facilitate VL joining [22]. With the absence of VL in nanobodies, these four hydrophobic residues are substituted for more hydrophilic amino acids (Phe37, Glu44, Arg45, and Gly47) [23–25] to avoid the exposure of such a sizeable hydrophobic region to solvent (Fig. 1). In addition to this substitution, residues adjacent to this interface have rotated their side chains without deforming the Cαbackbone to increase the VHH surface's hydrophilicity. Furthermore, the CDR3 domain of VHH folds over this interface to shield the amino acids formerly covered by the VL partner [26]. These alterations elucidate the augmented solubility of VHHs in comparison to the single VH domain and scFvs [27].

In VHHs, extension in hypervariable loops repays the loss of three VL CDRs and VH–VL combinatorial diversity. Extension of CDR1 and CDR3 provides a 600–800 Å2 antigen-interacting surface as offered by six loops from the VH–VL domain [24, 28]. Furthermore, the elongated CDR1, with mutational hotspots imprinted in the VHH germline, compensates for the VL partner's variability [29] while the somatic mutations in 28 and 30 residues of CDR1 are selected during the affinity maturation process to participate in antigen binding directly [30, 31]. Although the elongated CDR3 can extend into epitopes that are almost inaccessible for specific antibodies, the enlarged loop suggests broader flexibility, impeding antigen-binding entropically [24, 28]. To solve this issue, camellia VHHs evolved with an extra disulfide bond toward either the CDR1, CDR2, or FR2 [24, 28, 32]. All these structural features increase the paratope diversity and allow for a wide variety of geometrical loop structures that deviate fundamentally from the canonical loop structures defined for conventional antibodies and facilitate the orientation of the CDR3 toward the antigens [33, 34].

## Nanobody and scFv in properties

As a result of significant structural differences, scFv and Nb display distinct properties in vitro and in vivo. These different characterizations are investigated below in more detail (Table 1).

**Table 1:** A comparison between scFv and Nanobody in properties

Physiochemical properties	scFv	Nanobody
**size**	30-35 KDa 2×3 nm	12-15 KDa 1×2.5 nm
**Half-life in blood**	<1h	<<1h
**Preferred expression system**	Bacteria	yeast
**Water solubility**	**+**	**+++**
**Aggregation**	**++**	**−**
**Stability under harsh condition**	**+**	**+++**
**Tissue penetration**	**+**	**++**
**Paratope diversity**	**+**	**++**
**Cavity binding**	**−**	**+**
**High affinity**	**+**	**+**
**Ease of manipulation for enhance affinity**	**+**	**++**
**Complexity of library construction techniques**	**++**	**+**
**Nonspecific background binding**	**++**	**+**
**Intracellular functionality**	**+**	**++**
**Ease of expression**	**+**	**+++**
**Ability to concatenate**	**+**	**+++**

### Size

First and foremost, these two fragments have notable dissimilarity in their size, while scFv is almost twice the Nb size by about 30 kDa weight [35]. This smaller size facilitates VHHs genetic manipulation [36], and the presence of only three antigen-binding loops allows for easy enhancement of their intrinsic tendency to antigen [37]. Due to the renal filtration and degradation, the smaller size of VHHs also results in their short half-life in blood [38]. This feature can be helpful since it results in high tissue permeability but unfavorable because their molecular weight is below glomerular filtration cutoff size (65kDa) and makes problems in some clinical therapies requiring antibody circulation over extended periods.

This limitation has led to the development of half-life extension strategies that combine VHHs with additional molecules. One of the most popular ones is addition of stabilizing groups such as Poly-ethylene glycol (PEG) molecules that slow down blood clearance rate with high tumor or any other target site accumulation. Fusion with long-circulating serum proteins like albumin or albumin's specific binders, effectively increase the VHH half-life in the blood. Fc fusion can also stabilize them in blood while provoking the immune system to the target site. Furthermore, Fc or albumin fusion makes the antibody fragment size larger and implements FcRn-mediated recycling to increase the protein half-life in the blood [39].

### Solubility and Stability

As described previously, the substitution of four highly conserved hydrophobic amino acids for more hydrophilic residues in VHH leads to significant differences in properties between Nbs and scFvs. In scFvs, these four residues (V37, G44, L45, and W47) in FR2 form a hydrophobic interface to facilitate VH-VL joining. However, on the downside, this hydrophobic region lowers scFvs’ solubility, resulting in their high tendency for aggregation. The substitution of polar and smaller amino acid residues in this position (F37or Y37, E44, R45, and G47) makes them more hydrophilic and, consequently, more soluble than scFvs. Furthermore, this nonpolar to polar transition leads to the molecular and thermodynamic stability of VHHs in comparison to scFvs. Therefore, Nbs are more resistant to chemical denaturants and protease enzymes [40] and have higher stability under harsh PH or ionic strength [41]. This higher conformational stability also stems from the presence of an extra disulfide bond, which lowers the probability of heat-induced aggregation and limits VHHs flexibility [42–47]. Because of higher stability, they show high refolding efficiency, which means raising or lowering the sample temperature does not affect Nb conformation, i.e., it de-binds and binds to the target, respectively, without any aggregation or denaturation [48].

This rigidity in structure is a favorite property in the clinic since non-native protein aggregation is a common downside of antibody treatment, raising the immune response in severe cases [49, 50]. However, although in scFvs the hydrophobic interface between VL and VH dampens their stability, this two domain structures make them more flexible and more advantageous for some applications.

### Production process

Both scFvs and Nbs are generated from immune or naive libraries that will be screened to discover high-affinity fragments against our desired target. Despite significant similarities in library screening, the mechanism of library construction is less challenging for Nbs. VHH libraries are created from blood serum of either naive or immunized camelids. After mRNA isolation, cDNA is reverse transcribed by reverse transcription-polymerase chain reaction (RT-PCR). This pool of VHH sequences is applied to construct a library screened by versatile display technologies such as phage display to discover a specific VHH for any potential antigen [51]. Although the inaccessibility of a camel, dromedary, llama, or alpaca to immunize is a bottleneck, HCAbs generating transgenic mice, or commercial naïve or synthetic Nb libraries can circumvent this issue.

For scFvs, libraries are typically created from either naive or immunized murine or human. Similar to VHH, VH, and VL genes obtain from RT-PCR [52–55], but in contrast to Nbs, an extra step is required to connect VH and VL cDNAs through SOE-PCR. Pairing these fragments is a challenging step in the library construction of scFvs due to the low efficiency of this technique [56, 57]. Since the mispaired VH and VL developed by this method may not detect and bind to the target antigen [58].

After isolation of a specific scFv or nanobody, attaining a high expression yield is challenging, especially for scFvs. In scFvs, as discussed earlier, the intrinsic hydrophobic interaction between VH and VL domains leads to their higher tendency to aggregate. Therefore it is complicated to express them in various expression systems appropriately. Many methods should be employed to improve these fragments’ stability, such as loop grafting, altering specific positions in their structure, and random mutagenesis [59]. Nevertheless, the superior properties of VHHs, such as their high hydrophilicity lead to a less demanding production cycle with fewer steps [60].

Bacterial expression of scFvs can occur in the either cytoplasmic or periplasmic environment. Each of these systems has its own limitations for the proper formation of scFv. On the one hand, while chaperones and disulfide isomerases in periplasm are preferable for appropriate folding of scFvs [10], they lead to a lower production yield [61]. Furthermore, unpaired cysteine residues of scFvs can form covalent bonds with the periplasmic protein's cysteine, which results in aggregation [62, 63]. Adding a signal peptide such as Pel B should also be considered to guide scFv into the periplasm [61]. On the other hand, the reducing environment of the cytoplasm hampers disulfide bond formation, which leads to exposure of hydrophobic VH-VL patches to the solvent and production of insoluble aggregate forms of scFvs called inclusion bodies. These aggregated products have to be re-folded in one additional time-consuming, costly, and ineffective step with the need of denaturing agents like urea [64].

Another approach to lower scFv aggregation promoted by inter-domain hydrophobic interaction is the humanization of scFvs by replacing hydrophobic amino acids with hydrophilic residues to prevent accumulation. Although protein solubility has improved by this method, these replacements can also have minor effects on the antigen-binding affinity of the final scFv [65]. The single entity nature of VHH makes their production much easier besides scFvs. In VHH cytoplasmic expression, there are no such hydrophobic- interaction-related issues [66], which lower production costs [10].

scFvs are not efficiently expressed in the yeast expression system as well. Due to the higher hydrophobicity of scFvs, they cannot be produced with proper folding in Saccharomyces cerevisiae's Endoplasmic Reticulum (ER) [67]. Some extra refolding steps, such as co- or over-expression of chaperons, should be applied to overcome these limitations [68]. In contrast, S. cerevisiae is the best expression system for VHH [69], as organelles like ER or Golgi ensure proper disulfide bonds and glycosylation [70]. Because of these difficulties, E.coli remains the best host for scFv expression [10, 64, 68].

### Immunogenicity

One of the significant drawbacks of scFvs is their rodent origin, as the Hybridoma technique is only well developed for mice and rats and not for humans [71]. Murine VL and VH exhibit only 53% and 51% sequence identity, respectively, with corresponding regions in humans [72], while nanobodies display high sequence similarity with human VH (VH3 gene family) with ~75–90% identity correlated with their Low immunogenicity in clinical applications [73, 74]. As a result, the humanization process is more straightforward in VHHs. Even after the humanization of murine-derived scFv, the variable regions of scFv can elicit an anti-idiotypic response because eliminating critical residues in this region may affect antigen binding [75–77]. Also, scFvs’ engineering for reducing human anti-mouse antibody (HAMA) responses [78] will inactivate [79] injected scFvs and lessen their clinical effectiveness [80, 81], and allergic reactions will arise in repeated administration [78, 79]. Furthermore, humanization reduces the binding affinity of these fragments [10, 65], and CDR grafting may represent new immunogenic epitopes [75–77, 82]. In general, the humanization of murine-derived scFvs can overcome these immunogenicity problems to some extent but not entirely [59].

### Affinity

Although scFvs and Nbs provide similar affinity, they show a distinct preference for epitopes. Nbs have better access for grooves and clefts on the surface of antigens like ion channels [83], viral glycoproteins [84], or immune synapses [85], but scFvs prefer flat linear epitopes. These differences result from the longer CDR3 loop in Nb, allowing for a highly convex shape to access concave epitopes. Nbs also show a good affinity for flat epitopes suggesting that these fragments can form various interface complexes. Furthermore, nonspecific background binding is lower for nanobodies in comparison to scFvs [37].

## Nanobody and scFv in application

In recent years, by the rapid progress in antibody fragments engineering, the smaller size of scFv and VHH makes them suited for a broad range of applications ranging from therapy and diagnosis to research and exploration. Here the various utilizations of these prominent antibody fragments are discussed in detail to demonstrate which counterpart is more appropriate for each application.

### Therapeutic applications of Nb and scFv in various formats

**Fig. 2:**
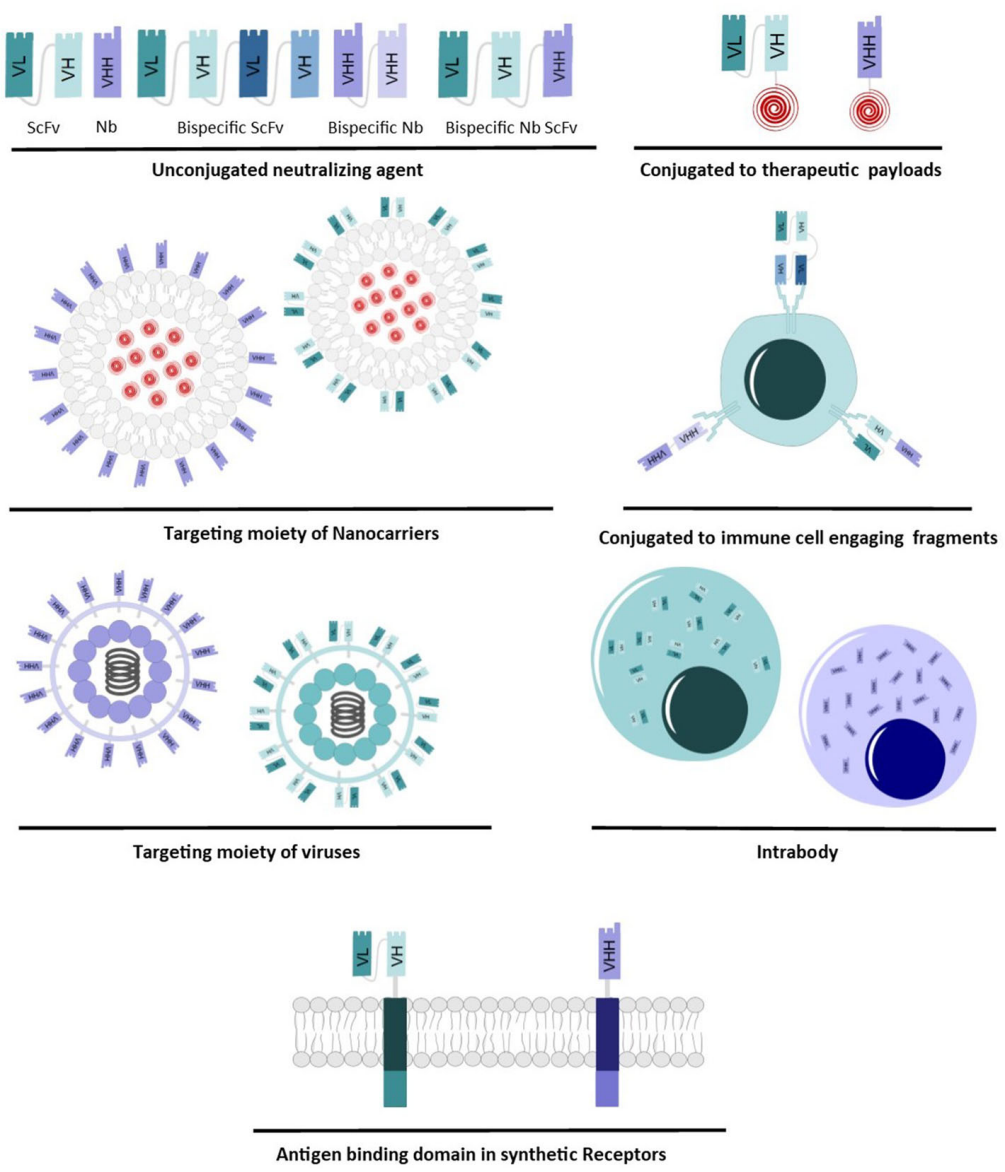
Different Formats of Nanobody and scFv in therapeutic applications

Development in targeted medicine has expanded treatment options, particularly in cancer therapy [45]. ScFv and VHH as high-affinity antibody fragments play an important role in targeted medicine [86] and are utilized in various formats (Fig. 2) as therapeutic options for several conditions.

#### Unconjugated neutralizing agent

ScFv and VHH can be utilized as neutralizing agents through direct binding and inactivation of foreign particles such as toxins and viruses [87] and cancer antigens or disease mediating cytokines and growth factors. These antibody fragments are administered naked in monovalent or multivalent format to block the function of their target or fused to an Fc region to increase serum half-life and trigger the immune system.

Nb is mostly a preferable candidate for this concern because of five main reasons. a) Their smaller size, resulting in perfect diffusion in tissues and extending neutralizing performance beyond the vessels. b) Higher flexibility and binding tendency enabling the bivalent and trivalent conjugation of Nanobodies and improving neutralization [88]. c) Their lower immunogenicity and more straightforward humanization process [89]. d) High stability that makes VHH production easier. e) Structural properties of VHHs that provide the power to target inaccessible epitopes [90].

Fatal toxins are a neglected but vital health problem all around the world. Plasma anti-venom serum (PAS) therapy is passive immunotherapy against toxins, with a rapid, effective response of direct antibody injection. Toxin neutralizers need to have rapid diffusion and clearance to provide enough power to identify and neutralize toxins with high tissue penetration [89] and then uptaken by kidneys in a shortened period. Therefore, antibody fragments such as nanobody and scFv are suitable for toxin inactivation, and scFvs were successfully applied in vitro. In a study done by Miethe and his colleagues, a neutralizing scFv-Fc inhibited the endopeptidase activity of botulinum neurotoxin [91]. In another paper, scFv was utilized to neutralize scorpion toxin Cn2 [88]. However, these fragments were not effective in vivo because of their high serum half-life [92]. In contrast, Nbs, with their smaller size and stronger binding affinity, have shown promising results in toxin inactivation in vivo [89]. Anti-scorpions toxin nanobody was able to protect mice more quickly than PAS therapy from Androctonus australis hector (Aah) scorpions [88]. In another study, despite the nanobody mixture failed to stop the venom lethality, the results proved the efficacy and usefulness of VHH developed against Bothrops atrox snake venom [93], which highlights the potential of VHH as anti-toxin agents.

Antibody fragments can neutralize viruses as well. In the last decades, one of the most successful approaches against viral agents was neutralizing monoclonal antibodies, which can suppress viral load partly through humoral immunity [94]. Nanobodies derived from Camelid and murine scFv are considered candidates for neutralizing viral agents because of their smaller size, higher solubility, and stability. Virus-neutralizing nanobodies have been developed against several animal and human virus families such as HIV-1 [95], human respiratory syncytial virus (hRSV) [96], and H5N1 Influenza [97]. The glycoprotein envelope of the virus canyons is filled with VHH, therefore the virus cannot conjugate with a co-receptor on the cell surface [98]. Bivalent and trivalent fragments are more efficient in identifying similar or different epitopes on the envelope glycoproteins, e.g., trivalent nanobodies against HIV [95] and hRSV [96] extended the neutralization breadth compared to the monovalent format. ScFvs are also utilized as viral neutralizing agents. Recombinant scFv has been utilized for targeting many viruses such as HIV [99], influenza [100], Porcine epidemic diarrhea virus (PEDV) [101], and HPV [102]. However, nanobody is more creditable because its smaller size provides the power to match and fill virus canyons more efficiently.

During the current SARS-Co-2 pandemic, anti-viral agents are one of the considerable therapeutic candidates. The receptor-binding domain (RBD) of the spike protein of the virus and the human angiotensin-converting enzyme 2 (ACE2) receptor on the cell surface are the key components in the viral entry that various forms of antibody fragments can target. Nanobodies [103] and scFvs are both appropriate candidates for RBD blocking, but Nbs are more common. The theranostic potential of camelid nanobodies in covid-19 infections is reviewed recently [104, 105]. Although a handful of scientific groups are working on finding the best nanobody set for SARS-cov2 neutralization [106–109], attempts are still enduring to discover a Recombinant scFv for this mission [110, 111]. Nevertheless, the superior properties of nanobodies for viral neutralization, such as lower production cost, the potential for aerosol delivery because of their high stability, and ease of multimerization put them forward in this race.

In terms of cancer immunotherapy, antibody fragments can inhibit tumor evasion by binding to the tumor itself or blocking the vital component for tumor growth and invasion. As growth factors play a critical role in angiogenesis, especially in the tumor microenvironment, blocking them or their cognate receptor can enhance tumor regression. Both scFv and VHH are utilized to bind and neutralize Vascular endothelial growth factor receptor 2 (VEGFR-2) [112, 113]. It is also demonstrated that the epidermal growth factor receptor (EGFR) blocked by a monovalent and bivalent nanobody called CONAN-1 could be a potential anticancer therapy [114].

Cytokine and chemokine inhibition is a potential therapeutic option for cancer and autoimmune disease [115]. Camelid VHHs and scFvs have been utilized for blocking several inflammatory and immunomodulatory cytokines as a treatment for rheumatoid arthritis [116] and chronic inflammation [117, 118], autoimmune inflammatory diseases [119] as well as cancer [86, 120], and stroke [121]. Cytokine- nanobody complex performs a sufficient neutralizing capacity when its half-life increases using albumin binding. It may act as a superior option in reduction of neuroinflammatory response in brain ischemia by high penetration, even crossing the brain-blood barrier [121]. Moreover, they can inhibit the enzymatic activity of their target protein, such as carbonic anhydrase (CAIX), by blocking their active site [122]. However, the efficiency of antibody fragment mediated immunotherapy can be further augmented by their conjugation with other therapeutic payloads discussed in the next section.

#### Conjugated to therapeutic payloads

Antibody fragments can be chemically or genetically conjugated to effector domains to deliver therapeutic payloads to a specific target, thereby reducing nonspecific toxicity to normal cells. Furthermore, this conjugation makes them larger, which results in increased circulation time in the blood [123].

One of the familiar domains that are genetically fused to these antibody fragments is toxins. Upon binding to the target cell, the complex is taken in through endocytosis, and finally, the toxin kills the desired cell- usually tumor or virally infected tissue [124]. Both scFv and nanobody have been utilized as an immunotoxin by conjugation with bacterial toxins like Enterobacter cloacae β-Lactamase [125] and Pseudomonas Exotoxin A [126, 127], or less immunogenic human origin cytotoxic elements such as TRAIL [128–130] and granzyme B [131].

Immunocytokines are another group of antibody fragment conjugates that can induce immune cell proliferation and anti-tumor activity in cancer immune therapy. Many studies demonstrated the effective function of these conjugates in specific anti-tumor activity [132–135]. The most popular cytokines delivered by antibody fragments are IL-2, IL-12, and TNF, whose systemic administration may lead to serious side effects [136].

Although scFv and nanobody can both serve as targeting agents for immunotoxins and immune cytokines, since tissue penetration is critical, especially in cancer, VHH is a better option for conjugation with larger fragments. However, the larger size of scFvs makes it suitable for conjugation with a smaller domain such as siRNA [137, 138]. Nevertheless, the superior physicochemical properties, easier humanization and better antigen recognition properties, and higher stability make camelid VHH preferable even in siRNA delivery [139].

#### Conjugated to immune cell engaging antibody fragments

Due to the critical role of the immune system in cancer, dual-specific antibody fragments can link between tumor and immune system components. Bite or bispecific T cell engagers are the most familiar form of these bispecific conjugates. By binding to CD3 with one of their recognition domains, they can activate T cell-mediated targeted tumor lysis. Bites are historically constructed from scFvs and scFv based the FDA now approves CD3 × CD19 BiTE to treat B-cell acute lymphoblastic leukemia [140]. However, camelid VHH can perform in each binding domain with a smaller size and higher modularity. Nevertheless, in recent studies, these two counterparts are mixed. For example, Harwood et al. created a novel form of T cell engagers named ATTACK by linking three anti-EGFR nanobodies with an anti-CD3 scFv as an ideal format for developing the next generation of T cell-redirecting bispecific antibodies [141].

Other immune system components such as NK cells γδTcells and APC can be redirected to the tumor site similarly by targeting their cognate marker. CD16 for engaging NK cells [142–144], T cell receptor (TCR) of Vγ9Vδ2 cells [145] and CD11b for attracting γδTcells, MHC-II or other specific surface proteins for targeting APCs [146, 147].

#### Targeting moiety of nanocarriers

Because of the binding specificity of these targeting agents, nanobodies and scFvs are broadly used in several drug delivery platforms to deliver their cargo to its specific location. High penetration, stability, pH-temperature resistance, and low aggregation are factors that shed light on VHH importance as a mediating delivery agent [148]. These delivery systems can overcome limitations of drug conjugate systems, such as poor chemical and enzymatic stability, solubility, rapid blood clearance, and adverse side effects to normal tissues. These nanocarriers can also release their cargo in more extended periods to decrease the frequency of drug administration [149]. Both VHH and scFv have been employed as a targeting moiety of nanocarriers such as liposomes [150–152], micelles [153, 154], albumin-based nanoparticles (NANAPs) [155, 156], and polymer-based NPS [157, 158]. But studies demonstrate the superiority of Nb in this regard [151]. In a study conducted by Oliveira et al., anti-EGFR-nanobody liposomes downregulate EGFR expression while its scFv based counterpart was unable to do so. The reason behind this effect is probably the sensitive structure of scFv that loses its proper folding in the acidic condition of lysosome after liposome internalization [151]. Furthermore, as these nanocarriers’ primary target is surface receptors that mediate cellular internalization [148], Nb, with its smaller size, is preferable to act as an antagonist for these receptors.

#### Targeting moiety of viruses

Viruses are eminent gene therapy agents applied for the treatment of various diseases and as a vaccine to induce an immune response against any desired protein. However, these highly efficient gene delivery systems cannot perform specifically by only infecting their target cells [159]. The binding specificity of antibody fragments is helpful to circumvent this issue. Many lentiviral and adenoviral vectors have been modified by either Nb or scFv [159–164], but the smaller size and high stability make it a better option in this regard. The superiority of Nb as a binding domain of adenovirus is perfectly investigated by Poulin et al. the result of this study demonstrate that the single domain antibody construct was efficiently incorporated into the Ad capsid and enhanced virus infection of cells expressing the targeted receptor while the scFv construct incorporated into the capsid at a very low level, insufficient to retarget virus infection [164].

#### Intrabody

Although most studies investigate the potential of antibody fragments in targeting extracellular antigens, most signaling pathways occur intracellularly [165]. Therefore targeting these intracellular factors may be effective in the treatment of various diseases, including cancer [166] and neurodegenerative conditions [167] such as Huntington's disease [168]. Intrabodies have been developed to express and operate within the cell and bind to an intracellular protein specifically. Because the reducing environment of cytoplasm is the main playground of intrabodies, stability under harsh conditions is the factor that makes Nbs outshine all the other antibody fragments as well as scFvs. However, the therapeutic potential of antibodies is hindered by the lack of an effective delivery system [165]. Viral delivery of intrabody coding sequence to the target cell is the most eminent method [167]. However, bacterial systems such as the type III secretion system (T3SS) of *E. coli* have been utilized to translocate the translated form of intrabody into their eukaryotic target.

#### Antigen binding domain in synthetic Receptors

Today with the impressive advancement in synthetic biology, a handful of synthetic receptors have been designed to sense and respond to extracellular signals in a programmable way [169, 170]. CAR (Chimeric antigen receptor) is the most popular form of these receptors that recently entered the clinic [171]. Similar to native receptors, the extracellular domain of these proteins is responsible for sensing extracellular factors. To improve the system's modularity and enable recognizing any desired factor, the extracellular part of these receptors is mainly composed of an antibody-derived fragment, such as scFv or nanobody. Although scFv is the most widely used form of the extracellular domain [169, 172], the superior properties of nanobody and its smaller size make it a preferable option in this position [172, 173]. In several studies, camelid VHHs have proven their efficiency as the antigen-binding moiety of CAR T cells [172–179]. Furthermore, the utilization of a bispecific nanobody-based construct in the receptor's extracellular domain broadens CAR T cell therapy specificity by recognizing two distinct antigens simultaneously [180].

Camellia VHH can serve as antigen binding domain in other less popular receptors as well. For instance, nanobodies have been utilized in the recently designed SynNotch receptor [181], GEMA [182], and C-STAR [183]. These new studies suggest that maybe in the future, nanobodies can take the position of scFvs in the extracellular domain of synthetic receptors due to their favorable properties such as high stability, smaller size, and low immunogenicity.

### Diagnostic applications of Nb and scFv

Although antibody fragments are well known for their therapeutic application, the binding specificity of these targeting agents suit them for several diagnosis applications. Not only in medical diagnosis but also environmental and food analysis applications. Their broad utilization in diagnosis is summarized in the following paragraphs.

#### Molecular imaging

Antibody fragments can serve as specific molecular probes for the detection of various disease-related antigens. Especially in cancer treatment, molecular imaging is critical for the early detection of tumor progression or monitoring the success or failure of the therapy in cancer patients. mAbs were the first high-affinity probes in molecular imaging, but their weak tumor penetration and high serum half-life lead to low contrast images with a low tumor to background ratio [184]. Smaller antibody fragments could circumvent this issue; however, Nb, as the smallest antibody-derived targeting domain, is superior in this regard. Compared with scFv and Fab, Nbs were shown to be promising probes for molecular imaging due to high tumor uptake, rapid blood clearance, low liver uptake, and high stability [185]. However, the rapid renal clearance of Nbs result in high signals in the kidneys and bladder, therefore they are not favorable for imaging at their nearby sites [37].

To date, several imaging techniques have been developed and applied for clinical application, and each of the antibody fragments is labeled with different agents to be visualized in that specific imaging technique [186–188]. Positron emission tomography (PET), single-photon emission computed tomography (SPECT) are the most popular imaging technique with many radionucleotides labeled nanobodies in clinical trials [189].

#### Immunoassays

إecause of the antigen-binding activity of immunoglobulins, the various format of the antibody-based immune assay, such as enzyme-linked immunosorbent assay (ELISA) and lateral flow immune assays (LFA) is designed for detection of various factors in medical diagnosis and environmental and food analysis. The mAb is the widely used targeting agent in various immunoassays, but high stability, lower production cost, and the ability to recognize the epitopes out of reach for larger fragments make Nbs a favorable immurement [184]. Nobody-based immune assays have shown promising results in detecting *T. solium* [190] and Trypanosoma [191], with better outcomes than their whole Ab counterparts.

To date, several formats of nanobody and scFv based immunoreagents have been utilized for detecting microorganisms, natural proteins, and chemicals in various specimens [192–201]. These detection systems are also packages as a point of care detection device known as a biosensor to enable portability, easier handling, and decrease production costs [202].

### Research applications of Nb and scFv

The application of antibody fragments is not limited to theranostics. These high-affinity binders are a proficient tool in research applications to understand the structure and function of proteins. Therefore, in this part, we mainly mention the use of Nbs and scFvs in fundamental research.

#### Protein visualization studies

Due to inefficient folding and chain assembly of scFvs, Nbs are better candidates to be stably expressed and then trace proteins in living cells [48, 203].i.e., reducing intracellular environment hampers proper folding and formation of disulfide bonds [204] in scFvs, results in their poor function and stability [205, 206], .therefore their use as intrabodies is restricted, and just a few of them have been used in fundamental biology researches. Nevertheless, Nbs do not show this limitation and can fold accurately inside the cells, which favors their use as a research tool for versatile applications over scFvs. , Furthermore adding a tag to label and visualize cytoplasmic proteins by vhhs is less challenging than scFvs as they show higher stability. Because of the single domain nature of Nbs, adding a tag does not influence their binding activity [207], while in scFvs, it is harder to add a tag without affecting the binding affinity

The first method for tracing any desired protein in developmental biology was fusing it with a fluorescent protein-like GFP. However, the intracellular expression of a protein and a fluorescent protein may influence the correct formation and function of the protein. To overcome such challenges, chromobodies were developed by fusing nanobodies with a fluorescent tag like RFP. Although target-specific chrome bodies can circumvent many challenges in this regard, the production of a specific nanobody for each target is labor-intensive. Hence targeting the fusion domains by chrome bodies enables the tracing of various proteins by a single nanobody. To date, many tracer nanobodies against small peptide fusions like SunTag and PTM have been developed; however, anti-GFP VHHs, termed (GFP binding proteins) GBPs, are extensively employed against GFP fusion proteins [208].

Another method of protein visualization used in fluorescence microscopy is imaging in a sandwich format utilizing a fluorescently labeled secondary antibody to detect the target bound Nb [209, 210]. However, in super-resolution microscopy, since it is more efficient to have a less distance between the target molecule and fluorescent label [211, 212], coupling organic dyes to GBPs can directly visualize any GFP labeled structure [213, 214]. In addition, chromobodies expressed in living cells can trace endogenous targets, therefore they are also employed in super-resolution imaging techniques [215]. In general, compared to scFvs, the smaller size of VHHs allows for a more accurate determination of the target's location in microcopy [216].

#### Protein function studies

Understanding the interactions of a protein to its surrounding environment is a critical step in studying the function of the desired protein. Antibody fragments as high-affinity binders can serve as a professional tool in studying protein-protein or even RNA-protein interactions. A study done by Sheetz et al. evaluated the function of human scFv for understanding the biogenesis of a subset of oncogenic microRNAs by using an anti-NCL scFv, they demonstrated that NCL is a critical protein in cancer biogenesis as it reacts with oncogenic microRNA. These antibody fragments can also serve as primary antibodies in ELISA [217, 218].

Another approach for study protein-protein interaction is GBP-based fluorescent-three-hybrid. In this approach, GBP is first coupled with an intracellular anchoring protein resulting in its fixation in the predetermined subcellular compartment. Then two target proteins which each one is fused with a fluorescent tag, interact with the central GBP binding protein. Therefore a GFP–RFP colocalization signal is produced at the location of GBP, which can be monitored to improve our understanding of protein-protein interaction [219].

Another method to understand the function of a protein is inactivation by degradation or interfering in the function, therefore the role of knockout protein in the pathway can be revealed in its absence. Unlike RNAi, Nanobodies, by their smaller size, can bind to the effector domain of desired proteins to defunctionalize that particular domain and enable the fundraising of its role in the process. In contrast, by RNA interference, the function of the whole protein would be disrupted, and the role of each domain will remain unclear [220].

Nbs can knock out their target protein by inducing protein degradation [221–224]. For instance, GBP nanobodies were combined with the F-box domain (which is a component of the SKP1–CUL1–F-box (SCF) ubiquitin E3 ligases complex) and so initiated polyubiquitination, which results in degradation of intracellular GFP-fused protein through proteasome [225]. Another approach is adding a PEST motif to Nb so that the target antigen will undergo a proteasomal degradation pathway [226].

Another step to studying a protein function is understanding its location of function. GBP nanobodies combined with monomeric RFP can localize GFP fusion proteins as they bind to GFP, and the resulting GFP–chromobody shows the location of GFP fusion proteins in nuclei, cytoplasm, or even membrane [227]. Nb is also a valuable tool to impose a new location for a protein of interest inside a cell. Nb fusion to localization domains redirect the POI to a new cellular compartment leading to its relocalization. In this manner, the role of location on protein function during animal development can be understood. If POI is tagged with a fluorescent molecule, this relocalization can also be monitored by fluorescent microscopy [219]. For instance, Nb coupled to KDEL peptide keeps its antigen inside the endoplasmic reticulum and coupling Nb with lamin molecule locate the antigen to the nuclear membrane [228]. Adding a tag to Nb can also immobilize the antigen is side the cell membrane and prevent its diffusion outside the cell [215, 229, 230]. By preventing the spread of secreted proteins, their effects on neighbor cells can be identified in developmental biology [231].

Nanobodies can also act as a scaffold that binds to two or more different targets in a non-overlapping manner. These target proteins can be DNA binding domains, and transcription factors as a ‘transcription device dependent on GFP’ (T-DDOG) [232] controls gene expression. This tool utilizes two GBPs, one of these nanobodies is equipped with a DNA-binding domain, and the other joined with the activation domain of the viral protein VP16. In GFP expressing living cells, these two Nbs come together in the presence of the GFP, leading to the desired gene expression.

#### Protein structure studies

Protein crystallography is one of the standard methods for understanding the structure of any desired protein. However, because proteins are highly dynamic, they are bound to crystallization chaperons for reduced conformational heterogeneity. Antibody fragments are superior among various binding partners since they can be engineered to target almost any desired protein [233]. In scFvs, the hydrophobic interface between VL and VH dampens their stability, and this two domain-structure make them more flexible than single-domain fragments like VHHs. This feature hampers their utility as a crystallization chaperon to understand an unknown and challenging protein [37].

In contrast, Nbs lower intrinsic flexibility show effective crystal formation, and it is easier to identify the conformation and mechanism of epitope binding by them rather than scFvs. VHHs can reveal protein conformations in each folding step, so transient intermediates can be determined even of highly dynamic protein [234] without inducing any out-of-native structure. To date, the structure of many proteins has been determined by VHHs, including high-value G protein-coupled receptor [235] and amyloid proteins in different pathological conditions [236, 237].

#### Affinity purification and Immunoprecipitation

Due to Nbs superior properties, including small size, monovalent mode, and easy directional immobilization to solid substrates, they can purify an increased amount of biomolecules in chromatography [24, 28, 238]. De Genest et al. developed VHHs against Glu–Pro–Glu–Ala (EPEA) in highly efficient affinity chromatography for any EPEA-tagged protein purification [239]. Nbs show lower nonspecific background binding than larger antibody formats like scFvs; besides, their high refolding efficiency and denaturation resistance allows for repeated column regeneration with only milder elution buffer, which later matters for sensitive targets [240]. Nanobodies can precipitate serum immunoglobins like IgG, so with their help, we can purify different types of antibodies from the blood. For example, Klooster et al. could purify human protein HSA and IgG from blood with the help of Nbs [241]. Furthermore, they are also perfect candidates for antibody-based slide, bead arrays [238], and chromatin immunoprecipitation with DNA microarray (chIP-on-chip) to discover new transcription factor-binding sites [242].

## Conclusion and future perspective

Antibodies as well-known targeting moieties are utilized for a handful of applications. The emergence of recombinant DNA technology enabled the design and construction of various antibody fragments, including Camelid nanobody and scFv. These two Ab fragments are the most widespread ones with a broad range of applications. Here we compared these fragments in structure and properties to investigate which of these antigen-binding domains is preferable for each application. In General, the higher stability, solubility, and lower production cost of Nb make it favorable in almost all applications, while its smaller size acts as a double-edged sword.

In applications that favor rapid clearance of targeting agents, such as molecular imaging or anti-venom therapy, the smaller size of nanobody is advantageous. While in other therapeutic utilizations that require persistence in the body, including neutralizing foreign invaders or targeting cancer, half-life extension strategies should be employed to increase the size of the targeting agent. On the downside, this size increment is against tissue permeability which is a critical property in cancer treatment. Therefore balancing the tradeoff between serum half-life and tissue penetration is necessary to achieve an optimized size for cancer antibody-based therapeutics.

Another issue that makes choosing between these two counterparts controversial is their preferred antigenic site. In other words, because of the structural difference between scFv and nanobodies, they prefer different epitopes while binding to the same antigen. Nbs are high-affinity binders for grooves and clefts, while scFvs prefer flat linear epitopes. Therefore, the antigen structure is an essential factor to decide which one is a better candidate for targeting the desired protein.

In spite of the superior properties of Nbs, scFvs are still dominant in the clinic with about ten FDA approved products and more than 80 ongoing clinical trials (Table 2). However, in less than 30 years since the discovery of HCAb, the superior properties of this single domain targeting agent make it a pioneer in the field of recombinant Ab engineering. In 2019 the first Nanobody entered the clinic and there are more than 27 Nb based drug candidate in clinical trials, waiting for FDA approval (Table 3). Nevertheless, because of the long history behind scFvs and other fragments derived from conventional Ab, nanobodies still have a long way to become a dominant player in recombinant antibody market.

**Table 2:** Some examples of various formats of scfvs in the market or clinical trails

Format	Antibody fragment	Target	Disease	Clinical trials	Phase	Status	Last update / approval date	Sponser
Unconjugated neutralizing agent	Brolucizumab	Vascular endothelial growth factor A (VEGFA)	neovascular age-related macular degeneration (AMD)	NCT03386474	3	FDA approved	Oct 2019	Novartis
	Emicizumab	Factor VIII	Hemophilia A	NCT02795767	3	FDA approved	Oct 2018	Hoffmann-La Roche
	Dinutuximab	GD2	neuroblastoma	NCT03098030	2&3	FDA approved	Apr 2015	United Therapeutics
Conjugated to immune cell engaging antibody fragments	Blinatumomab	CD19	Lymphoblastic Leukemia	NCT02393859	3	FDA approved	Mar 2018	Amgen
	Duvortuxizumab	CD19, CD3E	B-cell Malignancies	NCT02454270	1	Terminated	Dec 2018	Janssen Research & Development
Targeting moiety of nanocarriers	SGT 53	transferrin receptor (TfR)	Solid Tumors	NCT02340156	2	terminated	Mar 2021	SynerGene Therapeutics
	SGT-94	transferrin receptor (TfR)	Solid Tumors	NCT01517464	1	completed	Apr 2017	SynerGene Therapeutics
	MM-302	HER2	Breast Cancer	NCT02213744	2&3	terminated	Jan 2017	Merrimack Pharmaceuticals
Antigen binding domain in synthetic Receptors	Ciloleucel Yescarta	CD19	Diffuse large B-cell lymphoma (DLBCL)	NCT03391466	3	FDA approved	Oct 2017	Gilead
	Tisagenlecleucel Kymirah	CD19	Non-Hodgkin Lymphoma	NCT03570892	3	FDA approved	Aug 2017	Novartis
	Brexucabtagene	CD19	Relapsed/Refractory Mantle Cell Lymphoma	NCT02601313	2	FDA approved	Jul 2020	Gilead
	Lisocabtagene Maraleucel	CD19	Non-Hodgkin, B-Cell, Large B-Cell lymphoma	NCT03483103	2	FDA approved	Feb 2021	Celgene
	idecabtagene vicleucel	CD19	Multiple Myeloma	NCT03361748	2	FDA approved	Aug 2021	Celgene

**Table 3:** Different format of Nbs in clinical trials or market

Format	Antibody fragment	Target	Disease	Clinical trials	Phase	Status	Last update / approval date	Sponser
Unconjugated neutralizing agent	ALX-0081 Caplacizumab	von Willebrand Factor	Thrombocytopenic Purpura	NCT02553317	3	FDA approved	Feb 2019	Ablynx
	ALX-0061 Vobarilizumab	Interleukin 6 receptor (IL6R)	Systemic lupus erythematosus	NCT02437890	2	Completed	Feb 2019	Ablynx
			Rheumatoid Arthritis	NCT02287922	2	Completed	Aug 2019	
	ALX-0651	CXCR4	Healthy Volunteers	NCT01374503	1	Terminated	Apr 2012	Ablynx
	PF-05230905	CXCR4	Healthy	NCT01284036	1	Completed	Jan 2013	Ablynx
	ALX-0171	respiratory syncytial virus (RSV)	RSV infection	NCT02979431	2	Completed	Oct 2019	Ablynx
	ALX-0761	IL17A, IL17F and IL17A/F	Psoriasis	NCT03384745	2	Completed	Aug 2021	Avillion
	MSB0010841	IL17A/F	Psoriasis	NCT02156466	1	Completed	Jan 2017	Merck
	ATN-103 Ozoralizumab	TNF	Rheumatoid Arthritis	NCT01063803	2	Completed	Apr 2016	Ablynx
	KN035	PD-L1	Breast cancer Hepatocellular carcinoma	NCT04034823	2	Not yet Recruiting	July 2019	3D Medicines (Sichuan)
	KN044	CTLA-4	Advanced Solid Tumors	NCT04126590	1	Recruiting	July 2021	Changchun Intellicrown Pharmaceutical
	M6495	ADAMTS-5	Osteoarthritis, Knee	NCT03583346	1	Completed	Jan 2020	Merck
	LMN-101	Flagellin filament protein	Campylobacter Infections	NCT04182490	2	Not yet recruiting	Aug 2020	Lumen Bioscience
	VHH batch 203027	Rotavirus	Rotavirus Diarrhoea	NCT01259765	2	Completed	Aug 2011	International Centre for Diarrhoeal Disease Bangladesh
	n3088 n3130 Colchicine	SARS Cov2	COVID-19	NCT04322682	3	Completed	Jan 2021	Montreal Heart Institute
	TAS266	DR5	Solid tumors	NCT01529307	1	Terminated	Dec 2020	Novartis
	BI 836880	VEGF/Ang2	Neoplasm Metastasis	NCT03697304	2	Recruiting	Aug 2021	Boehringer Ingelheim
Conjugated to therapeutic payloads	L-DOS47	CEACAM6	Pancreas cancer Lung adenocarcinoma	NCT04203641 NCT03891173	2	Recruiting	Apr 2021	Helix BioPharma
Antigen binding domain of Synthetic receptors	TC-210 T Cells	Mesothelin	Mesothelioma Cholangiocarcinoma Ovarian Cancer Non Small Cell Lung Cancer	NCT03907852	1&2	Recruiting	June 2021	TCR2 Therapeutics
	BCMA CAR T cell	BCMA	Myeloma	NCT03664661	1	Unknown	Sep 2018	Henan Cancer Hospital
	CD19/20 bispecific CAR T cell	CD19 and CD20	B-Cell Lymphoma	NCT03881761	1	Recruiting	Mar 2019	Henan Cancer Hospital
	αPD1-MSLN CAR T cell	MSLN	Colorectal cancer Ovarian cancer	NCT04503980	1	Recruiting	Aug 2020	Shanghai Cell Therapy Group
Molecular imaging	131I-SGMIB Anyi-HER2 VHH1	HER2	Breast cancer	NCT02683083	1	Completed	Aug 2019	Precirix
	68GaNOTA Anti-HER2 VHH1	HER2	Breast cancer	NCT03331601	2	Recruiting	Feb 2021	Universitair Ziekenhuis Brussel
	99mTc-NM-02	HER2	Breast cancer	NCT04040686	1	Recruiting	Dec 2020	Shanghai General Hospital
	99mTc-Anti-PD-L1	PD-L1	Non-small cell lung cancer	NCT02978196	1	Completed	Aug 2020	Shanghai General Hospital
	99mTc MIRC208	HER2	Cancer	NCT04591652	NA	Recruiting	Oct 2020	Beijing Cancer Hospital
	68GaNOTA Anti-MMR VHH2	Macrophage Mannose Receptor (MMR)	Breast cancer Head and Neck Cancer Melanoma Carotid Stenosis Atherosclerosis of Artery	NCT04758650	2	Recruiting	Feb 2021	Universitair Ziekenhuis Brussel

## Data Availability

Not applicable.

## Abbreviation

Ab: Antibody
Nb: Nanobody
scFv: Single-chain variable fragment
Ig: Immunoglobulin
Fab: Fragment antigen-binding
Fc: Fragment crystallizable
VH: Variable heavy chain
VL: Variable light chain
V-NAR: Shark variable new antigen receptor
CDR: Complementary determining regions
FR: Framework region
HCAb: Heavy chain only antibody
IgNAR: Immunoglobulin new antigen receptor
FcRn: Neonatal Fc receptor
